# Rapid changes in hydrostatic pressure as a probe for correlating function of purified proteins with their measured activity in living cells

**DOI:** 10.1007/s12551-025-01331-9

**Published:** 2025-07-15

**Authors:** Daniel P. Mulvihill, Michael A. Geeves

**Affiliations:** School of Natural Sciences, University of Kent, Canterbury, CT2 7NJ UK

**Keywords:** Hydrostatic pressure, Cell biology, Proteins

## Abstract

Hydrostatic pressure (HP) has long been used to perturb protein and membrane structures and to alter their interactions with binding partners in a fully reversible manner. HP has also long been used to perturb molecular structures in living cells, where it can alter cytoskeleton dynamics and cellular signalling pathways and to stall cell division in a wide variety of cell types. HP can be applied and removed in a fraction of a second and is transmitted through tissue at the speed of sound; thus, rapid changes in HP can be very useful to correlate the behaviour of isolated macromolecules with the same molecules within living cells. Despite its usefulness, HP has not found wide use among researchers, mainly because of the need for specialist equipment. This largely reflects the use of high HP (≥ 1000 atmospheres) by the majority of practitioners. While these high pressures have provided insights into protein denaturation, membrane reorganisation, and sterilisation of bacteria and viruses in medicine and food, here we will focus on the uses of moderate HP (< 200 atmospheres) where the engineering and safety issues are less significant. At these lower pressures, HP alters the water shells at molecular interfaces. We outline here the background of the methods used and some of the simple adaptations required to laboratory equipment to allow HP studies and give some examples of its use for studying isolated proteins and the same proteins in living cells.

## Why use pressure?

The interface between biophysics and cell biology has been, and remains, one of the most exciting areas of molecular science. One of the major challenges in molecular cellular biology is the direct correlation of molecular and cellular events. While there has been significant progress in modulating gene expression and fluorescence labelling to follow localisation within cells, tools to directly perturb molecular interactions in the time scales of interest in a non-invasive, fully reversible way are somewhat lacking. Hydrostatic pressure (HP) is one way to perturb processes in a cell without an impact on cell viability. Applications of high-hydrostatic pressures (i.e. 1000 atm/100 MPa^‡^ or more (footnote: ^‡^NB we use atm and MPa interchangeably as both are widely used in the literature where 10 atm = 1.01 MPa) have been used to great effect to disrupt molecular and cellular processes. But high HP can cause irreversible changes to proteins and membranes, and thus impact cell viability. In contrast, moderate HP changes (i.e. 100 atm) are fully reservable and benign to proteins, organelles, and cells alike, having no observable impact upon cell viability. This benign effect on cell viability contrasts with the effects of HP on the physiology of multicellular organisms, which cannot survive without specialist adaptations. The current world record for free diving by humans is only ~ 100 m (https://thesaltsirens.com/current-freediving-records/) where HP reaches ~ 10 atm. The HP discussed here, ~ 100 atm, is that experienced on diving to a depth of 1000 m, a depth routinely accessed by several whale species (Helbo and Fago 2012). In contrast, single-cell and simple multicellular species can adapt to life in deep ocean trenches at 10,000 m where pressure reaches 1000 atm. Such organisms are called piezophiles (Scheffer and Gieg 2023) and while the adaptations required by such organisms are fascinating, they will not be discussed further, and the reader is referred to the specialist literature (Scheffer and Gieg 2023).

In addition to the relatively benign effect of moderate HP on cells and macromolecules, such HP is more technically straightforward to deal within the cell research laboratory environment. The low compressibility of water means a pressure chamber at 100 atm poses no more danger than a standard laboratory HPLC system. In fact, a standard microscope coverslip can support a 100-atm pressure difference provided the diameter of the window is small. Importantly, HP of 100 atm, therefore, has the potential for use with high-resolution fluorescence live cell imaging. Thus, limiting HP to only 100 to 200 atm opens up new opportunities for molecular research. At the same time, the potential for effects at yet lower pressure (i.e. ≤ 20 atm) has not yet been systematically explored.

The application of HP to protein folding provides an illustration of many of the features that make this method so versatile. The observation that HP could induce protein unfolding and denaturation was made in 1914 (Bridgeman 1914) and has been used since to destabilise proteins in the food and pharmaceutical industries (Baldelli et al. 2024) and to explore the dynamics and thermodynamics of protein folding and unfolding using rapid changes in HP (for review see Silva et al. 2001; Winter 2013). However, these approaches often use high pressures (> 1000 atmospheres/100 MPa) to cause major changes in protein structure. Here we are concerned with more moderate effects of pressure which perturb equilibria without complete denaturation of a protein or damaging cellular structures. We will outline the theory for pressure perturbation of equilibriums at modest pressures, before giving some examples of what has been achieved to date.

## Theory

All molecules in the solution are surrounded by a hydration shell, with the packing of water being more ordered around charged atoms or hydrophobic surfaces than in bulk solvent. This ordered water has a larger volume than bulk water (as in ice), and if HP is applied, then some of the bound water will be displaced in order to reduce the volume. In the case of small molecules like acetic acid H_3_C_2_OOH, then ionisation alters the water structure with more

$$\begin{array}{c}{H}_{3}{C}_{2}OOH\rightleftharpoons {H}^{+}+{H}_{3}{C}_{2}OO\\ \begin{array}{cc}V& V+\Delta \end{array}\end{array}$$

ordered water bound to the ions H^+^ and H_3_C_2_OO^−^ than to the non-ionised parent molecule. Such an ionisation occurs with ~ 10 cm^3^.mole^−1^ increase in volume (+ ΔV). Application of HP then results, via *Le Chatelier’s principle*, in the equilibrium shifting to the left to reduce the overall volume (Davis and Gutfreund 1976).

For a small change in the equilibrium constant (ΔK), the standard thermodynamic relationship applies:

1 $$\Delta K/K=-\Delta V\Delta P/RT$$

where *K* is the equilibrium constant, -Δ*V*° is the molar volume change for the reaction, Δ*P* is the applied HP change, *R* is the gas constant (= 82 cm^3^atm·mol^−1^ K^−l^), and *T* is the temperature in Kelvin. For an equilibrium ionisation such as acetic acid (above) with a -ΔV° of 10 ml/mole, a change of HP of 100 atm would result in ΔK/K = 0.04 (10·100/82·293 = 0.042). That is, a 100 atm change results in a small, 4% change in the equilibrium constant.

Similar effects, but smaller, can be seen in the exposure/burying of hydrophobic moieties. It should therefore be apparent that conformation changes in macromolecules, protein folding or ligand binding to proteins which result in changes of ionisation of side chains, and burying/exposure of charged or hydrophobic groups will be sensitive to pressure changes. Typical ΔV° values for protein denaturation are − 40 to − 100 ml/mole (chymotrypsin and myoglobin respectively), while the addition of tubulin to a microtubule has a ΔV° of − 26 to − 50 ml/mole (values taken from (Davis and Gutfreund 1976)). It should be noted that water itself and biological structures are relatively incompressible in this pressure range (water compressibility is ~ 0.46% at 10 MPa, (Weast and Astle 1979).

## Note on methodology

Full details of the pressure jump apparatus used for optical measurements on proteins and the experimental design are given in (Pearson et al 2002) and are based on an original design by (Clegg and Maxfield 1971). The protein sample is held in an optically polished sapphire ring (internal volume 50 µl) which allows absorbance and fluorescence measurements in the visible and near UV spectral regions. The top and bottom of the cell are sealed by a thin flexible kaptan membrane. A piston placed against the lower membrane is activated by a piezoelectric crystal stack which can move ~ 40 μm in ~ 0.1 ms. This can apply up to 200 atm HP to the sample and be maintained for several minutes or returned to 1 atm after times as short as 1 ms. The system allows multiple up and down pressure jumps to be applied repeatedly, and the combined data set was then averaged to improve the signal quality.

## Two examples of the use of HP to perturb equilibria of proteins in solution

### Protein folding

**Fig. 1:**
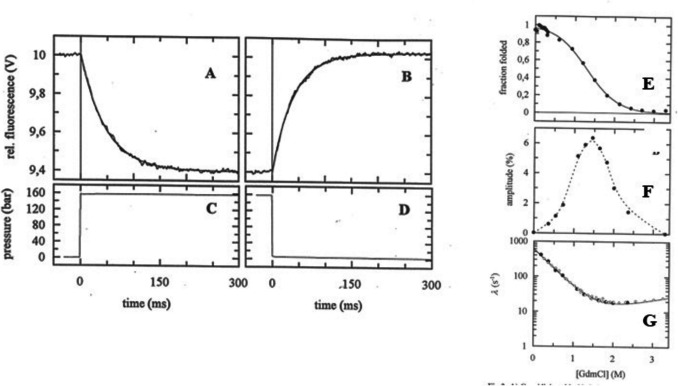
Pressure induced folding/unfolding of Cold Shock Protein (CSP). Protein fluorescence changes (**A** and **B**) induced by the pressure jumps (**C** and **D**) on 12 -μM Cold Shock Protein in 1.6 M GdmHCl, pH 7.0, 20 °C. The transients are the average of four pressure jumps on the same sample. GdmCl concentration dependence of **E** fraction folded, **F** amplitude of the folding transient, and **G** reciprocal relaxation time constant, λ, of the transient. Reprinted with permission from “Microsecond Folding of the Cold Shock Protein Measured by a Pressure-Jump Technique” Maik Jacob, Georg Holtermann, Dieter Perl, Jochen Reinstein, Thomas Schindler, Michael A. Geeves, and Franz X. Schmid. *Biochemistry* 1999 *38*, 2882–2891. 10.1021/bi982487i. Copyright 1999, American Chemical Society

The effect of moderate HP depends upon the position of an equilibrium as shown in Fig. 1 in which 160-atmosphere rapid pressure steps (up and down from 1 atm) were applied to Cold Shock Protein (Jacob et al. 1999). Rapidly increasing pressure (Fig. 1A and C) results in an exponential decrease in tryptophan fluorescence (relaxation time ~ 40 ms, change in fluorescence ~ 6%) as buried side chains are exposed to solvent. Reducing HP back to 1 atm (Fig. 1B and D) results in complete recovery of the signal with a similar relaxation time. The amplitude of the signal change depends upon the position of the Folded (F) $\leftrightarrows$ Unfolded (UF) equilibrium. Figure 1E plots the fraction of folded protein as a function of GdmCl concentration as measured from the steady-state protein fluorescence. Figure 1F plots the amplitude of the HP-induced signle change as a function of GdmCl concentration and has the form of a bell-shaped curve.The maximum amplitude of the pressure-induced transient occurs at the mid-point of the unfolding transition where [F] = [UF] and no detectable signal change occurs at the two extremes where the protein is > 90% folded or > 90% unfolded. This is a great advantage for working with proteins in cells as it means that pressures in this range are unlikely to cause significant destabilisation of the native structures. Plotting the observed reciprocal relaxation time (λ) vs [GdmCl] shows the familiar chevron plot (Fig. 1G) where λ is the sum of the two rate constants,

2 $$\lambda ={k}_{f}+{k}_{uf}$$

3 $${K}_{eq}=\left[UF\right]/\left[F\right]={k}_{f}/{k}_{uf}$$

and both rate constants are dependent upon the [GdmCl]. k_f_ dominates at low [GdmCl] where the CSP is predominantly folded, while k_uf_ dominates at high GdmCl concentration.

**Fig. 2:**
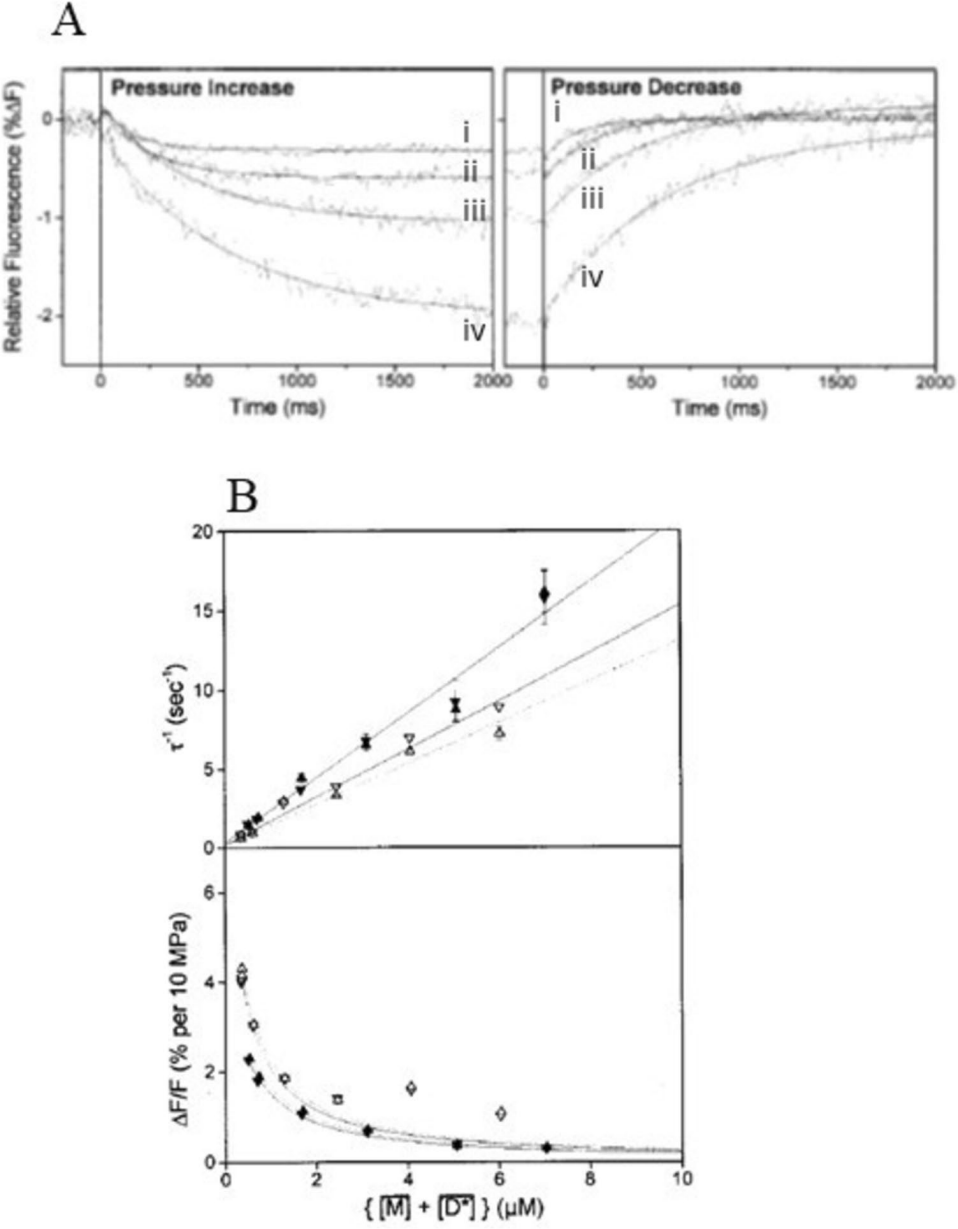
Pressure induced fluorescence transients of an equilibrium mixture of muscle myosin subfragment 1 (S1) and mantADP. **A** Observed transients showing the effect of changing concentrations of skeletal muscle S1 and mant-ADP after a 6-MPa pressure jump on a solution of skS1 with mant-ADP. The best-fit single exponentials are shown superimposed and the data shown are: (i) 4 µM S1, 1.0 µM mant-ADP, average of 10 transients; (ii) 2.5 µM S1, 1.0 µM mant-ADP, average of 5 transients; (iii) 1.2 µM S1, 1.0 µM mant-ADP, average of 3 transients; and (iv) 0.7 µM S1, 0.6 µM mant-ADP, average of 3 transients. **B** Concentration dependence of relaxation parameters for mixtures of mant-ADP with skeletal or smooth muscle myosin S1. The dependence of the reciprocal relaxation time (τ^−1^) and amplitude of the transient on the calculated free concentrations of S1 and mant-ADP, with skS1 (filled symbols) and smS1 (open symbols). The results of decreasing pressure are indicated by down pointing triangles whereas the results of increasing pressure are indicated by upward triangles. NB no significant change in τ^−1^ for jumps up to 6 MPa or down from 6 MPa. Reprinted with permission from “A novel pressure-jump apparatus for the microvolume analysis of protein–ligand and protein–protein interactions: its application to nucleotide binding to skeletal-muscle and smooth-muscle myosin subfragment-1” (2002) Pearson DS, Holtermann G, Ellison P, et al. Biochem J 366:643–651. 10.1042/BJ20020462 Copyright 2002, Springer

It should be noted that in Fig. 1A, the relaxation is observed at 160 atm while in Fig. 1B, the relaxation is at 1 atm, yet there is little difference in the value of λ. This is quite common for HP-induced relaxation times using HP changes in this range (see, for example, upper panel of Fig. 2B which plots the relaxation times for both up and down HP jumps). Studies of the effect of pressure on reaction rates (analogous to studies of the temperature dependence of reaction rates) normally require a much larger pressure range.

### Ligand binding to protein

A ligand binding to a protein can often be perturbed by a moderate change in HP, but like the protein folding reaction, the equilibrium has to be poised with near equal concentrations of free protein and bound to the ligand to detect a significant change in the concentration of bound ligand. This is illustrated in Fig. 2 for the binding of a fluorescent analogue of ADP (2′(3′)-O-(N-methylanthraniloyl)-ADP; mant-ADP) to the motor domain of muscle myosin (known as subfgragment 1) (Pearson et al. 2002).

$$M+mant-ADP\rightleftharpoons M.mant-ADP$$

The rapid pressure change of 6 MPa, up or down, induces a 0.2 to 2% change in total mant-ADP fluorescence, and the transient is well described by a single exponential (Fig. 2A). The amplitude and reciprocal relaxation time (τ^−1^) depend upon the concentrations of myosin (M) and mant-ADP in the equilibrium mixture (Fig. 2A transient i-iv; see figure legend for details). A plot of reciprocal relaxation time (τ^−1^) vs. the free concentrations of M (myosin subfragment 1) plus mant-ADP is linear

4 $${\tau }^{-1}=\left(\left[M\right]+\left[mant-ADP\right]\right)\cdot {k}_{on}+{k}_{off}$$

and the slope and intercept of the best fit line define the rate constant for association (k_on_) and dissociation (k_off_) respectively (Fig. 2B top panel). The observed amplitude (ΔF/F) is maximal at low concentrations, ~ 0.2 µM, close to the value of the estimated dissociation equilibrium constant which is k_off_/k_on_, and decreases as the concentrations increase (Fig. 2B lower panel). The amplitude is expected to decrease at lower concentrations than those shown in Fig. 2, but as the total fluorescence also decreases, this becomes limiting. Experimental details of the measurements are available in the figure legend, and the associated publication gives a fuller description.

## HP perturbation of complex protein assemblies

**Fig. 3:**
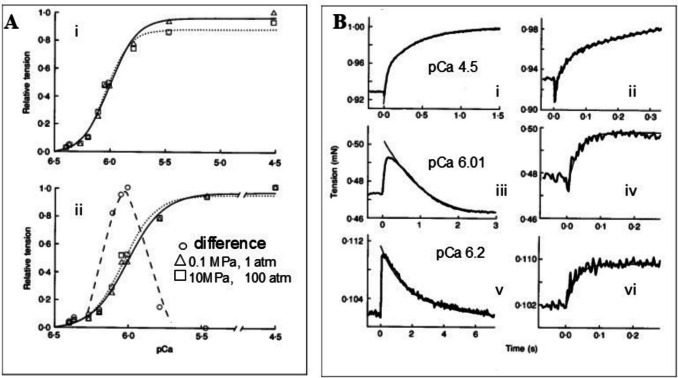
Influence of pressure on contracting skinned fast muscle fibres from rabbit psoas muscle. **A** The pCa (-log [Ca]) vs tension relationship at 1 atm (0.1 Mpa, triangles, solid line) and 100 atm (~ 10 Mpa, squares and dotted line). In **A**, the tension is normalised to the maximum at high calcium at 1 atm. In **B**, the same tension data is normalised to the maximum at high calcium in each case individually. Circles and the dashed line represent the difference between the 1 and 100 atm plots and appear as a bell-shaped plot with a maximum difference at ~ pCa 6.0. **B** Tension transients following a pressure drop from 100 to 1 atm in ~ 0.1 ms at three calcium concentrations (i and ii pCa 4.52, iii and iv 6.01, v and vi 6.2). The same data is shown on two time scales (i, iii, v slow, ii, iv, vi fast) to illustrate the two components of the transients. There is an instantaneous drop in tension in phase with the pressure change, which diminishes at lower calcium, and two exponential phases, both in the same direction at pCa 4.5 and in opposite directions at pCa 6.01 and 6.2. Reproduced with permission from “Contractile activation and force generation in skinned rabbit muscle fibres: effects of hydrostatic pressure” Fortune NS, Geeves MA, Ranatunga KW (1994). The Journal of Physiology 474:283–290. 10.1113/jphysiol.1994.sp020021. Copyright 1994, Wiley

A similar result is also seen for HP jumps on a more complex mixture of proteins. In Fig. 3, a single muscle fibre (10 mm × 0.1 mm dia) is held at a fixed length between two steel pins, one of which is attached to a force transducer (Fortune et al. 1994). The muscle fibre has had its outer membrane removed by treatment with detergent and this allows the fibre to be bathed in different solutions. In the presence of both ATP and calcium, the fibre contracts and holds a steady tension. As calcium is increased from 0.3 µM (pCa 6.5) to 30 µM (pCa 4.5), the steady tension increases and a fit to the Hill equation gives the mid-point as 0.98 µM (pCa 6.01, Fig. 3A). Repeating the measurement at 10 MPa HP results in a ~ 10% drop in tension at high calcium concentration. Replotting the two curves when scaled to the same endpoint (Fig. 3B) reveals a small shift in the mid-point to 0.91 µM. This could be considered to be a negligible change in the mid-point but analysis of the transient following the rapid release of pressure from 10 to 0.1 MPa shows the presence of two components. There is a fast component, complete in ~ 0.2 s with τ^−1^ independent of calcium concentration and an amplitude which is proportional to the steady-state tension (i.e. it decreases with calcium concentration).

This is followed by a slower component with a τ^−1^ that increases with calcium concentration and an amplitude which has a bell-shaped dependence on pCa with the same maximum (pCa = 6.0) as in the difference plot in the lower panel of Fig. 3A (open circles). This indicates the maximum amplitude for the slow transient occurs when TnC is 50% saturated with calcium. This slow phase is therefore consistent with a simple calcium binding reaction where the maximum amplitude of the transient occurs when there is an equal concentration of calcium bound and calcium-free TnC.

Deciphering the origin of the two components of the transient requires other experimental approaches not appropriate for this review. But in brief, the slow component is similar to a pressure-induced displacement of calcium from isolated troponin C (Pearson et al. 2008), the calcium binding protein on the muscle actin filament that must bind calcium to allow myosin access to its binding site on actin and muscle to contract. The amplitude of the fast component is proportional to the steady tension, which is consistent with this being part of the tension generation mechanism, i.e. the myosin motor. The value of τ^−1^ increases as the concentration of phosphate in the medium increases, which is consistent with this being the tension generating event in the actin myosin ATPase cycle, which is coupled to the release of phosphate from ATP. Fuller details are available (Fortune et al. 1991).

Thus, studies of isolated proteins have revealed that moderate high pressure (100–200 atm) can induce protein unfolding and dissociation of ligands from proteins and disrupt protein–protein interactions. Crucially, the pressure-induced change in the equilibrium constant for the reaction depends upon the value of the equilibrium constant, or more strictly on the free energy change for the reaction. Stable protein structures or tightly bound binding partners show minimal perturbation of the equilibrium position at these moderate pressures. This makes moderate pressure changes ideal for perturbing macromolecular complexes or living cells, where only poised equilibriums or steady-state systems will show significant sensitivity to HP. Thus, signalling pathways, for example, may be affected by relatively small changes in HP, leaving most other systems unperturbed. A second feature of pressure changes is that the pressure is transmitted through water at the speed of sound and will travel through cells within a tenth of a millisecond. Each of these features suggested to us that HP changes should be useful to explore the dynamic molecular events in living cells using the power of modern high-resolution microscopy. This approach would extend many current studies that use higher pressures to fix cells and then observe at 1 atm.

## High-pressure microscopy

HP has been established as a way to reversibly impact cell division of cells for quite some time now (Marsland 1938). In a series of now considered seminal experiments, Ted Salmon and colleagues followed disruption of cell growth and morphology of live mammalian (HeLa) cells subjected to HP of up to 400 atmospheres while under the microscope. When these samples were subsequently processed for immunofluorescence, researchers discovered an accumulation of cells in mitotic arrest, brought about by pressure-induced depolymerisation of microtubules (Salmon 1975a, 1975b; Salmon et al. 1976). These results are consistent with in vitro microscopy studies where researchers used bright, fluorescently labelled tubulin to show that high pressures promoted a linear-proportional rate increase in microtubule depolymerisation (up to 2000 atm) (Nishiyama et al. 2010). Further studies have subsequently been undertaken to correlate pressure-induced changes in cell morphology with the changes in gene expression within mammalian cells (Okamoto et al. 2021).

In a series of studies, Nishiyama and colleagues provided a further example of how HP can be used to modulate protein dynamics in vivo. When studying flagellar movement in *E. coli*, they found that a pressure of 800 atm induces arrest of bacterial flagellar movement (Nishiyama and Sowa 2012), while higher pressures (approaching 1200 atm) triggered a fully reversible switch in the direction of rotation of the flagellar molecular motor in living bacteria (Nishiyama et al. 2013). In a series of fascinating experiments, *E. coli* cells were subjected to very high HP on the microscope stage, allowing the researchers to follow speed and direction of movement of rotating flagellar motors. They discovered a switch from counterclockwise to clockwise rotation at different temperature-dependent pressure thresholds. Thus, by modulating pressure, the researchers were able to change the interactions between regulatory proteins to directly control the activity and movement of this microbial molecular motor.

While the above in vivo experiments relied on transmitted light microscopy modalities, the advances in imaging and labelling technologies allow organelle and molecular dynamics to be followed in live cells while subjected to pressure. In 2006, Frey and colleagues used a custom HP system that allowed the use of fluorescent dyes to follow DNA morphology and mitochondrial membrane potential to study the impact of subjecting live mammalian (Raji) cells to HP (up to 3000 atm) for periods of up to 10 min (Frey et al. 2006). Another example was the use of fluorescent calcium sensors to follow movement through ion channels. While pressure does not affect intracellular calcium levels to induce changes in cytoskeletal organisation (Crenshaw and Salmon 1996), ion channels are extremely sensitive to pressure, and calcium levels spike more than 50% higher at 100 atmospheres compared to normal pressure. Pressure studies have provided mechanistic insight into cell–cell signalling. This has been demonstrated to great effect in the motile algae, *Chlamydomonas reinhardtii*, where pressure impacts calcium channels to increase activation of microtubule motors within flagella (Yagi and Nishiyama 2020).

With the advent of fluorescent protein fusions, it has become routine within the cell biology field to follow the dynamics of individual proteins and organelles in real time within living cells. Indeed, there are numerous examples where cytoplasmic fluorescent protein-based biosensors have been used to monitor changes in internal pressure using modified YFP-based pressure sensors (Watanabe et al. 2013) or metabolic components (Bourges et al. 2020). Over the last few years, advances have been made in the development of monomeric fluorescent proteins with high quantum yields (see https://www.fpbase.org for a regularly updated, wide range of examples) and reduction in costs of ≥ 90% quantum efficiency (QE) image capturing devices. These developments make the real-time study of intracellular molecular dynamics at higher pressure accessible to all cell biologists with a sense of adventure.

High-pressure in vivo fluorescence microscopy allows the opportunity to study the many unicellular and metazoan cell systems that have evolved pressure-induced changes in metabolic and physiological behaviour, such as marine organisms or cells within tumours. Examples include the in vivo analysis into the molecular basis of the graded response of ciliary photoreceptors to pressure to regulate movement (Bezares Calderón et al. 2024) or bioluminescence (Bao et al. 2023) of deep-sea marine organisms, or shifts in wavelength sensitivity (Timpmann et al. 2021) and electron coupling energy (Jalviste et al. 2020) in light-harvesting photosynthetic bacteria. Similarly, it can be a powerful tool in medical research for characterising the underlying triggers of the pressure response in tumour cells and angiogenesis (Mammoto et al. 2022), as well as studying pressure-sensitive signalling pathways implicated in oncogenesis (e.g. TORC) (Uemura et al. 2020).

However, we have recently shown that moderate pressures (100 atm) can bring about changes in the morphology and molecular interactions within cells. We found it impacted cytoskeletal function and cell cycle regulation in different yeast cells (Brooker et al 2018). Thus, although high pressures have their place, it would appear that much lower pressures can perturb delicately poised equilibria within a cell and therefore provide a less technically challenging approach to study biological problems.

## Technical considerations for HP experiments

At the moderate pressures highlighted here, application of pressure is relatively straightforward. Then, 100–200 atmospheres can be applied using a hand cranked water or oil pump and released through a simple manual valve to reduce pressure in < 1 s (Davis and Gutfreund 1976; Coates et al. 1985). Equally, pressure can be applied using standard HPLC pumps and plumbing components and has the advantage that constant pressure can be maintained for several hours, or even days if required (Geeves and Ranatunga 1987; Knight et al. 1993; Brooker et al. 2018). The disadvantage is that corrosive media and buffers can limit the lifetime of pump components, but this can be avoided by separating the experimental sample buffer from the water of the pump by a deformable membrane without limiting the pressure range or time resolution of the system. A more sophisticated approach is the use of piezo crystal stacks to apply pressure. Provided the total volume is kept small (< 0.05 ml), then pressures of 200 atm can be applied and maintained indefinitely. Application and release can also be completed in 100 µs (Clegg and Maxfield 1971; Pearson et al. 2002).

**Fig. 4:**
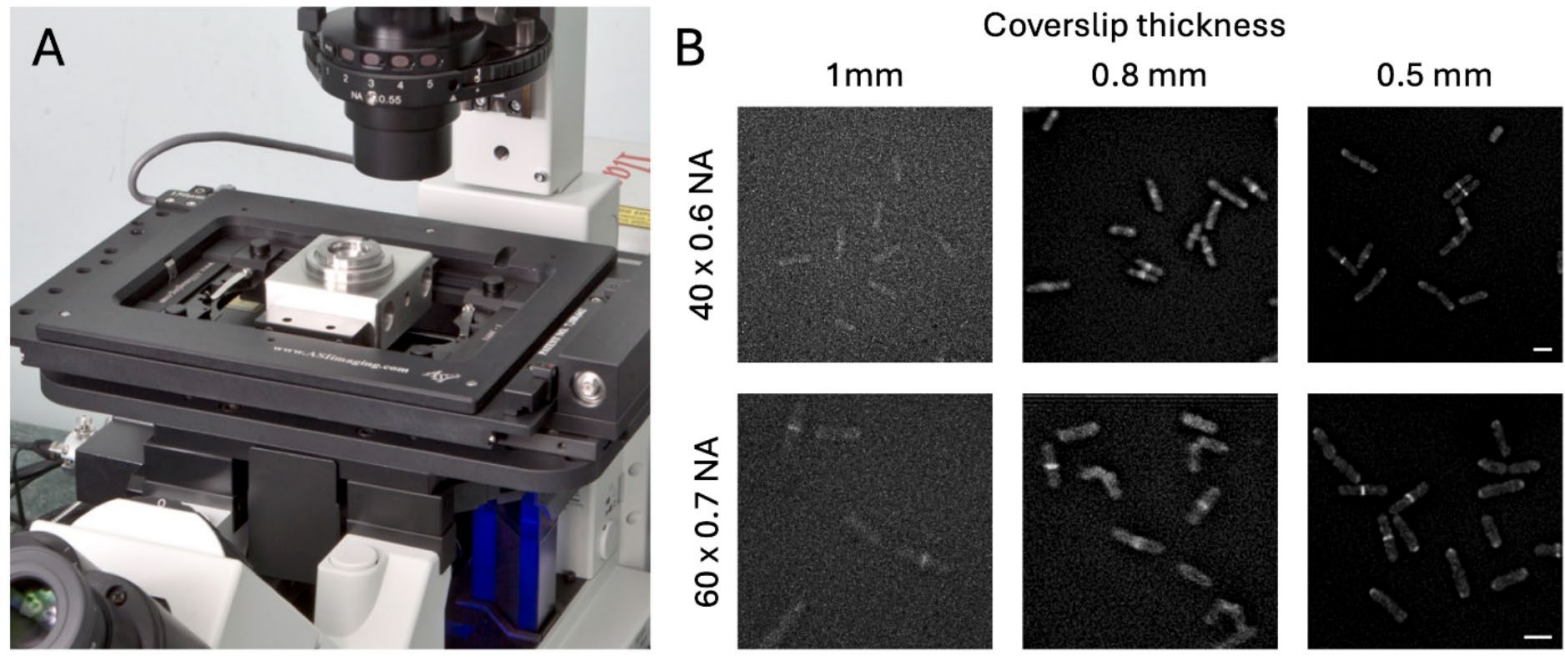
Fluorescence microscopy of live cells at 100 atmospheres pressure. **A** A pressure chamber can be easily integrated into the light path of an existing live cell imaging system (see Brooker et al., 2016 for details on HP chamber design). **B** Impact of coverslip thickness and lens numerical aperture upon image quality. Live fission yeast cells (*Schizosacchaormyces pombe*) expressing GFP labelled Calmodulin (Cam1-GFP) were mounted with lectin onto different thickness glass coverslips (0.5, 0.8, and 1.0 mm) and imaged at 100 atm using × 40 0.6 NA (upper panels) and × 60 0.7 NA (lower panels) air lenses. 0.5-mm glass coverslips can tolerate pressures of 100 atm for sustained periods and allow close to the Nyquist limit of resolution for the lens. Scale bars: 10 µm

A secondary consideration is the design of the window which is needed for the observation of the sample at these pressures. The most common signals are the usual protein spectroscopic signals of fluorescence and absorbance. These visible and near UV signals can be observed through sapphire windows, which are able to stand pressures well in excess of 200 atm for a diameter of several mm. Many other signals have been used, including X-rays (Knight et al. 1993; Möller et al. 2016), EPR (Grosskopf et al. 2024), and NMR (Caro and Wand 2018), but each requires specific window design. A major advantage of the HP ranges discussed here is that even quite common materials, like glass and plastic, can be used, provided the window diameter remains small, at a few mm, with recognition that the windows will fatigue and therefore need regular replacement. We will discuss window design further in the section on microscopy and in Fig. 4.

Similarly, as long as some basic factors are considered, it is possible to easily adapt a standard fluorescence light microscope to allow organelle, cytoskeleton, and even protein dynamics to be followed at the relatively modest pressures (i.e. ≤ 200 atm) required to impact cell growth, morphology, and division (Fig. 4). The need to capture images through thick coverslips, necessitated by higher pressures, makes imaging with usable resolution extremely challenging. The thicker the coverslip (typically ≥ 3 mm thick in studies highlighted at start of this section), the lower the resolution, due to light refraction through the material, be that glass, quartz, or sapphire. However, with some careful planning and design, imaging with sub-micron lateral resolution at 100 atm pressure is readily achievable (Fig. 4).

Designing a coverslip holder with the smallest feasible observation hole that also matches the objective lens shape not only minimises the surface of the coverslip that is exposed to larger pressure differences but also helps minimise the “working distance” between the lens and sample (Brooker et al. 2018). The working distance is another important factor in determining the maximum possible resolution for image data, as the closer the sample is to the lens, more light can be captured through it. This is one of the reasons why total internal reflection fluorescence microscopy (TIRF) lenses, which are used to capture data within a few hundred nanometres of the surface of the thinnest of coverslips, have the highest numerical apertures (NA—defines light gathering ability and resolution of lens).

A range of microscope lenses with the required 2 mm working distances required for a basic well-designed HP chamber are available from the major microscope manufacturers. Typical × 60 magnification 2 mm working distance air lenses, with an NA of 0.7, allow you to resolve a GFP labelled protein with a maximum 444 nm resolution, while × 60 2 mm working-distance water-dipping lenses (NA ~ 1.1) increase the potential maximum resolutions up to 282 nm at the GFP wavelength. By shaping the coverslip support to match the lens, we have found it is possible to maintain 100 atm pressure for sustained periods (i.e. overnight) using coverslips of only 0.5 mm thickness, thus minimising light diffraction and allowing you to approach the maximum possible resolution for a lens (Fig. 4). The resolution can be improved further by using 3D deconvolution software to reconstruct 3D z-slice datasets, which removes background fluorescence signal, originating from outside the focal plane, to enhance image quality and resolution. Thus, the design of the coverslip support and lens choice is crucial in defining the pressure limit and resolution of an HP-imaging system and will allow the dynamics of the cytoskeletons, nuclei, and other organelles to be followed within a cell.

Once the chamber is designed and built, pressures can be easily applied by using manual hand-driven pumps, piezo motors, or even pumps salvaged from unwanted high-pressure chromatography systems. No off-the-shelf HP imaging systems are currently available, and researchers are required to custom design, build, and develop pressure chambers for their microscope set-up and experimental requirements; however, for reference, the design of the system used in this laboratory is described elsewhere (Brooker et al. 2018). The pressure system can be extremely simple to operate, and with only the most basic of programming knowledge, it can be controlled from a simple applet or integrated into your existing microscope control software. This allows a stable high pressure to be monitored and maintained over long-sustained periods (> 24 h), which can be rapidly reversed, allowing instant triggering of affected intracellular molecules and physiological response(s) within cells. One key point is that the pressures described and volumes required for the experiments here make the experiments safe with minimal risk and, therefore, do not require extensive safety precautions.

Using this approach, we have previously described the development of simple HP chambers, requiring only a modified stage insert to allow easy integration into an existing inverted microscope system (Fig. 4), with required no modifications to the optical light paths (Brooker et al. 2018). With a lens with appropriate working distance and numerical aperture and using 0.5-mm-thick coverslips, we were able to follow cytoskeletal dynamics at 100 atm for a sustained period. With this system, we observed no compression of red blood corpuscles, muscle fibres, or different unicellular yeasts. We also established that at 100 atm pressure, stress signalling pathways were not activated, indicating any delays or changes in cellular behaviour were due to changes in dynamic molecular interactions. We found culturing cells on the imaging chamber overnight at 100 atm pressure led to the accumulation of binucleate, aseptate cells, which synchronously underwent cytokinesis upon returning to normal pressure. Intriguingly, this disruption of cytokinesis is reminiscent of the phenotype observed in Marsland’s original 1938 study on sea urchin eggs (Marsland 1938). This system, and systems like it, is suitable for studying the impact of pressure on a wide range of cells and metazoan organisms.

## Summary

To date, researchers have only begun to scratch the surface of the questions that can be addressed using this powerful non-invasive, rapid induction (i.e. speed of sound) fully reversible physical perturbation. We have highlighted some key literature historical experiments and demonstrated what is currently achievable, yet the questions that can be examined with this type of system are almost limitless. It has been shown in both budding yeast and bacteria that mutations can be introduced into genomic loci to provoke pressure-sensitive mutant arrests (Funada et al. 2022; Gayán et al. 2023). Using these mutants with a fluorescent HP imaging system would allow the opportunity to follow the instant impact of perturbing specific molecular interactions upon the cell and its intracellular components. We restricted our work to 100–200 atm, but the effects reported here may respond to yet lower HP changes (i.e. ≤ 50 atm). Lower pressures make pressure cell design (thinner windows and or greater diameter windows) much simpler, so studying the impact of lower HP has upon cells remains an attractive opportunity to explore.

## Data Availability

No datasets were generated or analysed during the current study.
